# P-1040. Clinical Outcomes of Micafungin for Invasive Fungal Infections in the Obese and Nonobese

**DOI:** 10.1093/ofid/ofae631.1230

**Published:** 2025-01-29

**Authors:** Ryan Moran, Beth Cady

**Affiliations:** SSM Health St. Louis University Hospital, Palatine, Illinois; Southern Illinois University at Edwardsville School of Pharmacy, Hillsboro, Illinois

## Abstract

**Background:**

The efficacy and safety of echinocandins make them favorable first-line options for many fungal infections. Current guidelines do not recommend dose adjusting echinocandins for obesity. Increasing evidence suggests pharmacokinetic goals may be difficult to attain with standard echinocandin dosing in obese populations but clinical outcomes data is scarce. The purpose of this study is to investigate if obese patients given micafungin for invasive fungal infection (IFI) have worse outcomes compared to nonobese patients.

**Methods:**

This retrospective study compared obese and nonobese patients receiving micafungin for IFI. Primary outcome was favorable clinical response in obese patients compared to nonobese patients with IFI at the end of antifungal therapy. Inclusion criteria were age ≥ 18, suspicion of IFI, and receipt of micafungin for ≥ 72 hours. Doses other than micafungin 100 mg were excluded. Patients were placed into one of two groups. Patients with a body mass index (BMI) < 30 kg/m^2^ were assigned to the nonobese group and those with a BMI ≥ 30 kg/m^2^ were assigned to the obese group.

**Results:**

600 patients were evaluated resulting in 378 being included with 177 and 201 in the obese and nonobese groups, respectively. There were minor variations in baseline characteristics. The primary outcome occurred in 49% in the obese group and 51% in the nonobese group (p=0.76).

**Conclusion:**

These results do not show a difference in clinical outcomes using micafungin in obese and nonobese patients and do not support use of higher doses of micafungin in the obese. It is unknown whether there is truly no difference or if no difference was seen due to small sample size. Additional research is needed to further evaluate echinocandins and clinical outcomes in obese patients.

**Disclosures:**

**All Authors**: No reported disclosures

